# LAG-3-Expressing Tumor-Infiltrating T Cells Are Associated with Reduced Disease-Free Survival in Pancreatic Cancer

**DOI:** 10.3390/cancers13061297

**Published:** 2021-03-15

**Authors:** Lena Seifert, Ioana Plesca, Luise Müller, Ulrich Sommer, Max Heiduk, Janusz von Renesse, David Digomann, Jessica Glück, Anna Klimova, Jürgen Weitz, Marc Schmitz, Adrian M. Seifert

**Affiliations:** 1 Department of Visceral, Thoracic and Vascular Surgery, University Hospital Carl Gustav Carus, TU Dresden, 01307 Dresden, Germany; lena.seifert@ukdd.de (L.S.); max.heiduk@ukdd.de (M.H.); janusz.vonrenesse@ukdd.de (J.v.R.); david.digomann@ukdd.de (D.D.); jessica.glueck@ukdd.de (J.G.); juergen.weitz@ukdd.de (J.W.); 2 National Center for Tumor Diseases (NCT), Partner Site Dresden, 69120 Heidelberg, Germany; marc.schmitz@tu-dresden.de; 3 German Cancer Consortium (DKTK), Partner Site Dresden, German Cancer Research Center (DKFZ), 69120 Heidelberg, Germany; 4 Faculty of Medicine Carl Gustav Carus, Institute of Immunology, TU Dresden, 01307 Dresden, Germany; ioanaplesca.m@gmail.com (I.P.); luise.mueller1@tu-dresden.de (L.M.); 5 Faculty of Medicine Carl Gustav Carus, Institute of Pathology, TU Dresden, 01307 Dresden, Germany; ulrich.sommer2@ukdd.de; 6 Faculty of Medicine Carl Gustav Carus, Institute for Medical Informatics and Biometry, TU Dresden, 01307 Dresden, Germany; anna.klimova@mailbox.tu-dresden.de; 7 National Center for Tumor Diseases (NCT), Core Unit for Data Management and Analytics (CDMA), 01307 Dresden, Germany

**Keywords:** pancreatic cancer, tumor microenvironment, tumor-infiltrating T cells, ICOS, LAG-3, PD-1, VISTA

## Abstract

**Simple Summary:**

In light of the majority of pancreatic cancer patients not responding to current immune checkpoint blockade, alternative immunotherapeutic targets need to be identified. In this study, we employed multiplex immunofluorescence to investigate the expression of co-stimulatory and inhibitory receptors by tumor-infiltrating T cells in human pancreatic cancer. A comprehensive analysis of the receptor pattern on tumor-infiltrating T cells is essential for the development of new therapeutic strategies, as well as personalized immunotherapy, to identify patients who are likely to benefit from targeting specific immune receptors.

**Abstract:**

T cells are the predominant immune cell population in the pancreatic tumor microenvironment. High CD8^+^ and Th1-polarized CD4^+^ T cell infiltration is associated with prolonged survival in human pancreatic ductal adenocarcinoma (PDAC). However, the expression pattern of co-stimulatory and inhibitory receptors by PDAC-infiltrating T cells and their prognostic significance are not well defined. In this study, we employed multiplex immunofluorescence to investigate the intratumoral expression of the co-stimulatory receptor inducible T-cell co-stimulator (ICOS), the inhibitory receptors lymphocyte-activation gene 3 (LAG-3), programmed death 1 (PD-1), and V-domain immunoglobulin suppressor of T cell activation (VISTA) by tumor-infiltrating T cells (CD3) in a cohort of 69 patients with resected PDAC. T cells were enriched particularly within the stromal area and were highly heterogeneous across tumors. Further, T cells were associated with prolonged disease-free survival (DFS). However, LAG-3 expression by PDAC-infiltrating T cells was correlated with reduced DFS. Our study highlights the biological importance of LAG-3 expression by tumor-infiltrating T cells. LAG-3^+^ T cells may represent a novel prognostic marker and a particularly attractive target for immunotherapeutic strategies in PDAC.

## 1. Introduction

Pancreatic ductal adenocarcinoma (PDAC) has a poor prognosis, with fewer than 9% of patients surviving five years after diagnosis, and the outcomes have not improved significantly with new therapies over the past years [1,2]. Despite the efficacy of immunotherapeutic strategies in multiple solid tumors, including melanoma, kidney, bladder, and lung cancer, the results of early trials investigating the blockade of cytotoxic T-lymphocyte-associated protein 4 (CTLA-4) and programmed death-ligand 1 (PD-L1) in patients with advanced-stage PDAC have not demonstrated clinical benefits [3,4,5]. Immunotherapies targeting co-stimulatory and inhibitory receptors beyond CTLA-4 and programmed death 1 (PD-1) have entered clinical trials [6]. Accumulating evidence indicates that the tumor immune contexture, comprising the spatial organization, density, and functional orientation of tumor-infiltrating immune cells, plays a critical role in the clinical outcomes and responses to immune checkpoint blockade (ICB) in cancer patients [7]. T cells are the most prevalent immune cell type in PDAC, and the majority of resectable PDACs display intermediate to high levels of T cell infiltration [8,9,10]. CD4^+^ and CD8^+^ T cells tend to reside within the stromal area [11,12]. CD8^+^ T cells and Th1-polarized CD4^+^ T cells mediate tumor protection in murine PDAC and are associated with prolonged survival in human disease [13,14,15,16]. Co-stimulatory and inhibitory receptors are expressed differentially on T cell subsets and during specific stages of T cell differentiation. Further, they determine the functional outcome of T cell receptor signaling [17]. Inducible T-cell co-stimulator (ICOS; CD278) is an activating co-stimulatory receptor expressed by T cells [18]. Lymphocyte activation gene 3 (LAG-3), PD-1, and V-domain Ig suppressor of T cell activation (VISTA) are inhibitory receptors contributing to T cell exhaustion [19]. A potential mechanism of resistance to checkpoint blockade is the expression of multiple inhibitory receptors that may dampen T cell responses [20]. There is a limited understanding of co-stimulatory and inhibitory receptor expression in PDAC. To gain novel insights into their clinical relevance in PDAC, we employed multiplex immunofluorescence of tumor tissue from patients with resected pancreatic cancer.

## 2. Results

### 2.1. Tumor-Infiltrating T Cells Express Co-Stimulatory and Inhibitory Receptors in Pancreatic Cancer

**Figure 1:**
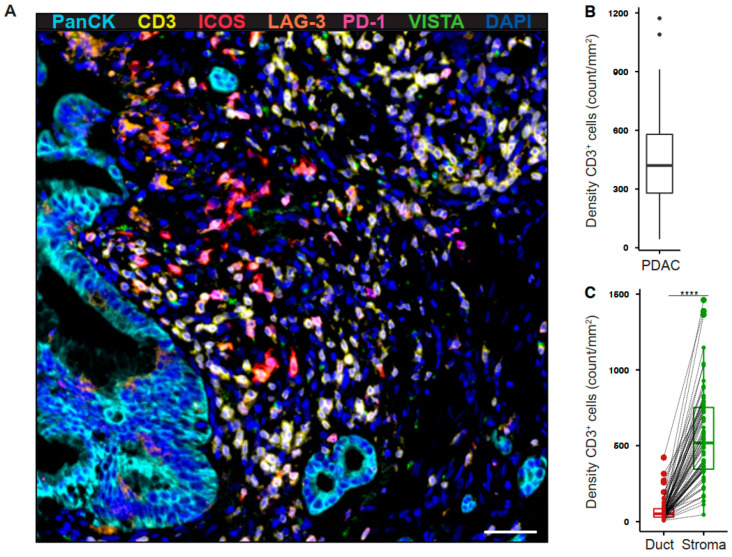
Tumor-infiltrating T cells reside within the stromal area in pancreatic cancer. (**A**) Paraffin-embedded human pancreatic ductal adenocarcinoma (PDAC) specimens were stained for PanCK (cyan), CD3 (yellow), ICOS (red), LAG-3 (orange), PD-1 (purple), and VISTA (green). Representative multiplex immunofluorescence image is shown (200×). Scale bar, 50 μm. (**B**) Quantification of T cell density in whole PDAC and (**C**) ductal (red) and stromal (green) tissue areas. Each point represents a single patient (total, *n* = 69). Dot plots and box-and-whiskers (plus min-max), median. Paired Wilcoxon test. *p*-values ≤ 0.05 were considered significant. ****, *p* < 0.0001.

**Figure 2:**
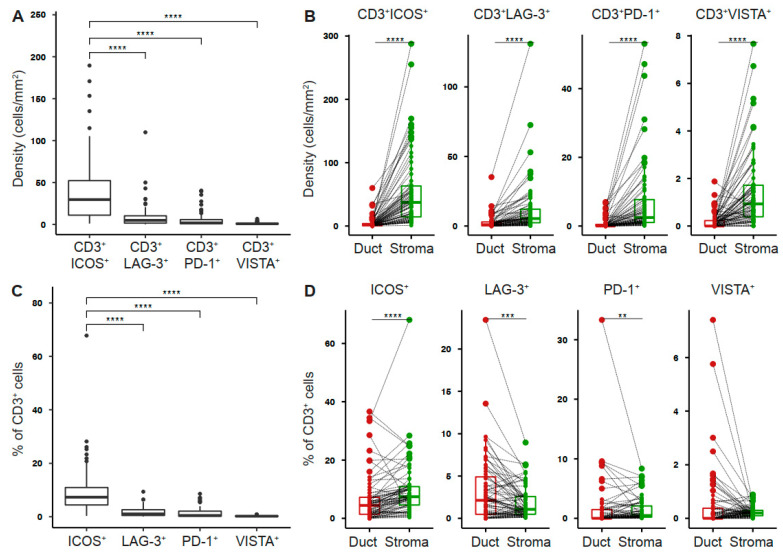
Tumor-infiltrating T cells express co-stimulatory and inhibitory receptors in pancreatic cancer. (**A**) Density of T cells stained positive for indicated receptor in whole PDAC and (**B**) ductal (red) and stromal (green) tissue areas. (**C**) Percentage of T cells positive for indicated receptor in whole PDAC and (**D**) ductal (red) and stromal (green) tissue areas. Each point represents a single patient (total, *n* = 69). Dot plots and box-and-whiskers (plus min-max), median. Paired Wilcoxon, and Kruskal–Wallis test and Dunn multiple comparisons test. *p*-values ≤ 0.05 were considered significant. **, *p* < 0.01; ***, *p* < 0.001; and ****, *p* < 0.0001.

To determine the expression profile of co-stimulatory and inhibitory T cell receptors in the pancreatic tumor microenvironment, we performed multiplex immunofluorescence for CD3, ICOS, LAG-3, PD-1, VISTA, and pan-cytokeratin (PanCK) (Figure 1A). The intratumoral density of T cells was highly variable across tumors (Figure 1B). However, T cells were most abundant in the tumor stroma (Figure 1C). ICOS^+^ T cells were the most prevalent population (41.87 ± 5.04 cells/mm^2^), whereas VISTA^+^ T cells were rare (1.13 ± 0.15 cells/mm^2^, Figure 2A). The density of receptor-positive T cells was significantly higher in the stromal as compared to the ductal compartment (ICOS 54.88 ± 7.00 vs 5.19 ± 1.2 cells/mm^2^, LAG-3 11.71 ± 2.35 vs 2.71 ± 0.61 cells/mm^2^, PD-1 7.03 ± 1.32 vs 0.71 ± 0.19 cells/mm^2^, VISTA 1.44 ± 0.19 vs 0.2 ± 0.04 cells/mm^2^, Figure 2B). Next, we examined the percentage of T cells positive for each receptor. 9.74% (± 1.16%) of PDAC-infiltrating T cells expressed ICOS, whereas a smaller proportion of T cells expressed LAG-3 (1.89 ± 0.23%), PD-1 (1.47 ± 0.24%), and VISTA (0.25 ± 0.02%, Figure 2C). The percentage of ICOS-, PD-1-, and VISTA-expressing T cells was significantly higher in the stromal compared to the ductal area, whereas the percentage of LAG-3-expressing T cells was specifically high in the ductal compartment (Figure 2D).

### 2.2. Intratumoral T Cell and PD-1^+^ T Cell Densities Are Associated with Increased Disease-Free Survival in Pancreatic Cancer

**Figure 3:**
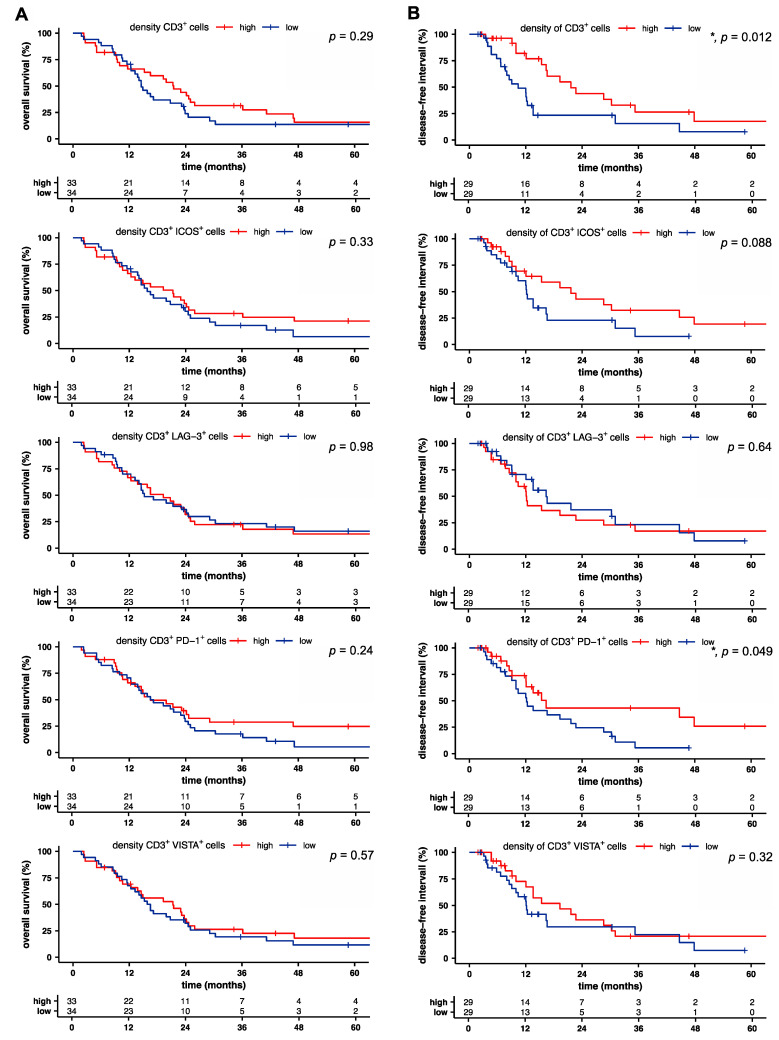
Intratumoral T cell and PD-1^+^ T cell densities are associated with increased disease-free survival in pancreatic cancer. (**A**) Overall and (**B**) disease-free survival of patients with pancreatic cancer, stratified by the median of indicated density. Tick marks indicate censored data. *p*-values were calculated using a log-rank test. *, *p* < 0.05.

We next sought to identify prognostic factors for overall (OS) and disease-free survival (DFS) based on T cell infiltration and the receptor expression pattern in pancreatic cancer. OS was independent of T cell infiltration and receptor expression (Figure 3A). However, univariate analysis revealed a significant association of prolonged DFS with higher T cell density (*p* = 0.012), increased density of PD-1-expressing T cells (*p* = 0.049), and a trend for the association with the density of ICOS-expressing T cells (*p* = 0.088; Figure 3B). Furthermore, a high distribution of ICOS^+^ T cells correlated with reduced DFS, whereas all other proportions and distributions of receptor-positive T cells showed no correlation with overall and disease-free survival (Figure S1A,B).

### 2.3. LAG-3^+^ T Cells Are Associated with Poor Disease-Free Survival

**Figure 4:**
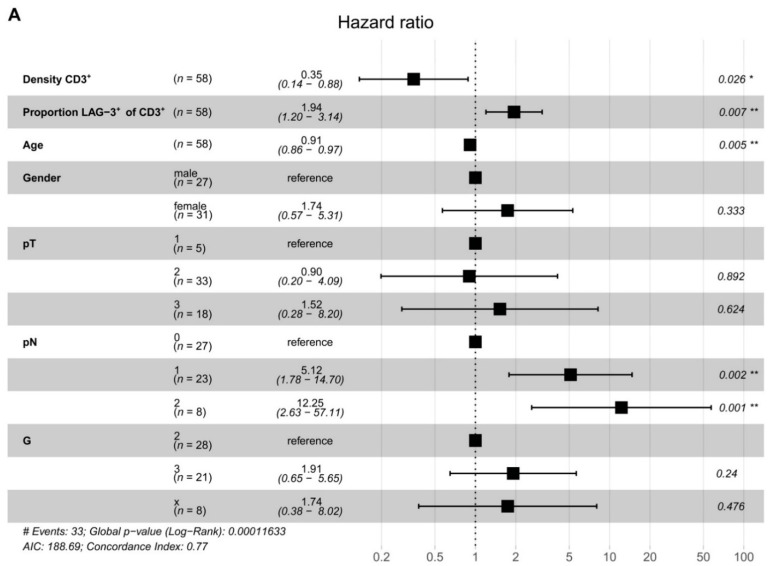
LAG-3^+^ T cells are associated with poor disease-free survival. (**A**) Risk of tumor recurrence in pancreatic cancer patients. Hazard ratios and 95% confidence intervals are shown. *p*-values ≤ 0.05 were considered significant. *, *p* ≤ 0.05; and **, *p* < 0.01.

To assess interdependence, we performed a Cox proportional hazard model (Figure 4A). Multivariable analysis showed that T cell density (*p* = 0.026) and the proportion of LAG-3^+^ T cells (*p* = 0.007) were independent prognostic factors for DFS in PDAC. T cell density was associated with prolonged DFS. Notably, the proportion of LAG-3-expressing T cells was predictive for reduced DFS. These data highlight the biological importance of PDAC-infiltrating T cells in general and emphasize the relevance of LAG-3 expression. LAG-3^+^ T cells may represent a novel prognostic marker for patients with PDAC.

## 3. Discussion

PDAC patients with high T cell infiltration and neoantigen qualities promoting T cell responses have shown improved survival [21,22]. However, except for microsatellite instable tumors, which account for less than 1% of PDAC, immunotherapeutic strategies have not demonstrated clinical benefits [23,24,25]. In light of patients not responding to current anti-CTLA-4 and anti-PD-1 treatment, alternative immunotherapeutic targets need to be identified in PDAC [3,4,5]. Little is known about the relative prevalence and distribution of co-stimulatory and inhibitory receptors in PDAC. Previously, we identified the immune ligand galectin-9 as a diagnostic marker for the detection of PDAC [26]. Tumor-infiltrating T cells showed upregulation of galectin-9 compared to T cells from matched blood. Here, we analyzed the composition and prognostic significance of T cells and their expression of ICOS, LAG-3, PD-1, and VISTA in 69 resected PDAC specimens. T cell infiltration was heterogeneous and highly variable across tumor specimens, consistent with a previous report [15]. We found that T cell density was significantly increased in stromal compared to ductal areas. Further, T cell density was an independent predictor of DFS. Notably, long-term survival has previously been associated with pathologic factors, such as lack of lymph node metastasis and tumor differentiation, as well as T cell infiltration and quantity and quality of tumor neoantigens [21,22,27,28]. ICOS and ICOSL binding lead to T cell activation and effector functions and, when sustained, induce suppressive activities, mediated by regulatory T cells [18]. We observed a high prevalence of ICOS+ T cells in PDAC with a trend towards prolonged DFS in patients with a high ICOS+ T cell density. It is possible that targeting ICOS may unleash an anti-tumoral T cell effector function. In colorectal cancer, high ICOS expression by activated effector T cells was correlated with improved survival [29]. The human ICOS antibody KY1044 is currently being investigated in solid tumors, including PDAC (NCT03829501). Targeting co-stimulatory receptors on T cells might synergize with checkpoint inhibitors to increase functional immune infiltrates specific for tumor antigens [20]. Further studies are needed to investigate the relevance of ICOS expression by T cells in PDAC. Antigen-specific and exhausted T cells typically express inhibitory receptors, including LAG-3, PD-1, and VISTA [30,31]. Our multiplex immunofluorescence analysis revealed a highly variable expression of these inhibitory receptors, which has also been observed in melanoma [32]. Further, we found no significant association of inhibitory receptor expression by T cells with tumor stage. The density of receptor-positive tumor-infiltrating T cells was unchanged with neoadjuvant chemotherapy compared to primary resected patients. Our data indicate a relevant role of LAG-3 expression by T cells for DFS and suggest that the presence of intratumoral LAG-3+ T cells may contribute to early disease recurrence in PDAC. LAG-3 expression leads to dampened CD4+ T cell activation, enhanced Treg suppressor activity, and decreased cytotoxic CD8+ T cell function [33]. LAG-3 expression has been demonstrated to be associated with reduced OS and DFS in hepatocellular carcinoma and in Epstein–Barr-virus-positive and MLH1-defective gastric cancer [34,35]. In head and neck squamous cell carcinoma, LAG-3 was upregulated on tumor-infiltrating T cells and correlated with reduced OS [36]. In bladder cancer, the abundance of LAG-3+ cells in the tumor stroma indicated an immunoevasive contexture and represented an independent predictor for poor OS [37]. Notably, elevated expression of LAG-3 by tumor-infiltrating lymphocytes in PDAC patients was previously detected along with increased PD-1 and CTLA-4 expression [38]. In line with our data, LAG-3 expression may be a potential mechanism of immune evasion in PDAC, especially since in non-small cell lung carcinoma (NSCLC) prominent LAG-3 expression by T cells was associated with insensitivity to PD-1 blockade [39]. Recently, we have shown a relevant role for PD-1 in the immune suppressive network of T cells from PDAC-draining lymph nodes [40]. Here, we observed prolonged DFS in PDAC patients with an increased density of PD-1 expressing T cells, suggesting a double-edged role of PD-1+ T cells. Notably, PD-1 expression has been demonstrated to identify T cells that recognize tumor-specific proteins [41,42]. Further, we found very little VISTA expression by PDAC-infiltrating T cells. In a recent study, VISTA was expressed by approximately 25% of tumor cells in PDAC and was significantly associated with prolonged OS [43]. T-cell-specific VISTA expression, however, showed no correlation with patient outcomes, suggesting that its expression by tumor and non-T cells might be more relevant in PDAC. However, a previous report showed a reciprocal correlation of VISTA expression and anti-tumor T-cell responses and cytokine production of tumor-infiltrating lymphocytes [11]. These observations emphasize the necessity of a comprehensive analysis of the receptor pattern on tumor-infiltrating T cells for personalized immunotherapy in order to identify patients who are likely to benefit from targeting specific immune receptors.

## 4. Patients and Methods

### 4.1. Patient Samples

The study cohort consisted of 69 patients with PDAC who underwent surgery in our department between 2008 and 2015. All patients consented to a protocol approved by the Ethics Committee of TU Dresden (No EK446112017) and the study was approved by the institutional review board of the Faculty of Medicine of TU Dresden. All tumor samples were formalin-fixed and paraffin-embedded, and a serial section was stained with hematoxylin and eosin for histologic evaluation by a trained pathologist. The clinical stages of tumors were determined according to the tumor-node-metastasis (TNM) classification system developed by the Union for International Cancer Control (UICC; Edition 8). Patient characteristics are shown in Table 1.

### 4.2. Multiplex Immunofluorescence

The Opal™ kit, together with the Vectra^®^ 3 automated quantitative pathology imaging system (both from Akoya Biosciences, Menlo Park, CA, USA), were employed to conduct tyramide signal amplification (TSA)-based multiplex immunofluorescence. This technology has been previously used by various research groups to simultaneously detect a wide range of molecules characterizing immune cells across different cancer entities [44,45]. One of the main advantages of this multiplex approach is that it allows for staining multiple markers on the same paraffin-embedded tissue section, irrespective of the species of the primary antibodies, thus enabling the visualization of multiple co-expressed molecules on the same cell. To design the multiplex panel for this project, we followed the typical workflow recommended by the manufacturer (Akoya Biosciences), which has also been employed, tested, and validated by other groups [46,47,48,49]. Thus, we first started with classical immunohistochemical staining of each antibody. Then, to check the staining quality and pattern, to select the best working primary antibody–fluorophore combination, and to adjust the dilutions of the reagents, uniplex staining for each individual marker using the Opal™ kit was performed. Subsequently, the markers of interest (ICOS, LAG-3, PD-1, and VISTA) were tested in duplex stainings with the anti-CD3 antibody. Finally, the additional markers were integrated one-by-one until a six-plex panel was created, while making sure that the pattern of each marker did not change from uni- to multiplex. For all the TSA immunofluorescence stainings, tissue deparaffinization and hydration were performed as described before [50]. For antigen retrieval, all tissue sections underwent a microwave treatment in AR9 buffer (Akoya Biosciences). Subsequently, the Opal^TM^ kit was used. In brief, an initial 10-min blocking step was followed by the incubation of the tissue sections with the primary antibody for one hour at room temperature. Then a horseradish peroxidase-conjugated secondary antibody (Akoya Biosciences or Thermo Fisher Scientific, Rockford, IL, USA) was applied for 10 or 20 min, respectively. Finally, a TSA fluorophore was added to the tissue sections for 10 min. A microwave treatment was performed afterwards for the stripping of the primary antibody, together with the secondary antibody. All the steps mentioned above, from blocking to stripping, were repeated for each primary antibody. In the end, all tissue sections were counterstained with spectral 2-(4-amidinophenyl)-1H-indole-6-carboxamidine (DAPI; Akoya Biosciences) for 5 min and coverslipped with fluoromount medium (SouthernBiotech, Birmingham, AL, USA). For the multiparametric stainings, primary antibodies directed against PanCK (clone AE1/AE3, 1:250, Thermo Fisher Scientific), CD3 (polyclonal, 1:75, Dako), ICOS (clone D1K2T, 1:75, Cell Signaling Technology), LAG-3 (clone EPR4392, 1:250, Abcam, Cambridge, United Kingdom), PD-1 (clone NAT105, 1:50, Abcam), and VISTA (clone SP344, 1:125, Abcam) were used, together with the TSA fluorophores 650 (1:900), 520 (1:75), 570 (1:75), 620 (1:175), 540 (1:100), and 690 (1:75, all from Akoya Biosciences), respectively. Visualization of the staining was performed using the Vectra^®^ 3 automated imaging system (Akoya Biosciences). First, all whole sections were scanned. Then, they were used to annotate the multispectral images (MSIs), covering a proportion of 25–50% of the tumor-containing area, in the Phenochart^TM^ software (Akoya Biosciences). The selected MSIs were scanned at a magnification of 200× and further used for analysis. The inForm^®^ software (Akoya Biosciences) was employed for spectral unmixing, using a library built from single stained tissue slides for each primary antibody–TSA fluorophore pairing, as well as for phenotyping. This latter step relied on a semi-automatic approach, in which an experienced user teaches the software to discriminate distinct tissue areas, segment the cells, and then individually phenotype each cell subset. For each marker, we created a different phenotyping algorithm, based on MSIs acquired from different patients, which we also tested for performance on another set of MSIs before applying it to the entire cohort. By creating a novel phenotyping protocol for each marker, we could reliably detect co-expressed markers on the same cells, while also having more flexibility for data processing. R software was used to handle and analyze the output data from InForm^®^.

### 4.3. Statistical Analysis

Data are shown in dot plots and box-and-whiskers graphs (min; max), median. The two-tailed paired or unpaired Kruskal–Wallis test and the Dunn multiple comparisons test were performed as applicable. The distribution was defined as the ratio of ductal to stromal density. Kaplan–Meier curves were used to visualize differences in overall survival (OS) and disease-free survival (DFS). OS was defined as the time between surgery and death for any reason. DFS was defined as the time between surgery and disease recurrence. Patients with 30-day or in-hospital mortality were excluded from the survival analyses. Median values of the cohorts were used as cut-offs. Significance was determined utilizing the log-rank test. Using a Cox proportional hazard model, we explored the hazard ratio of receptor expression, in combination with patients’ clinicopathological characteristics. All statistical analyses were performed using R software (survival, survminer package). *p*-values ≤ 0.05 were considered significant. *, *p* ≤ 0.05; **, *p* < 0.01; ***, *p* < 0.001; and ****, *p* < 0.0001.

## 5. Conclusions

In this study, we performed a comprehensive analysis of co-stimulatory and inhibitory receptor expression by PDAC-infiltrating T cells using multiplex immunofluorescence. Our data underscore the prognostic relevance of T cell density for prolonged DFS and further identify a negative prognostic role for LAG-3 expression by T cells in human PDAC. LAG-3^+^ T cells may not only represent a novel prognostic marker, but may also be a particularly attractive target for immunotherapeutic strategies in patients with pancreatic cancer.

## Figures and Tables

**Table 1:** Clinicopathologic characteristics of PDAC patients.

Variable	*n* = 69 *n* (%)
**Age**	
Median (Range)	68 (36–79)
**Gender**	
Male	33 (48)
Female	36 (52)
**pT Stage**	
1	7 (10)
2	39 (57)
3	21 (30)
Unknown	2 (3)
**pN Stage**	
0	32 (46)
1	26 (38)
2	11 (16)
**pM Stage**	
0	68 (99)
1	1 (1)
**UICC Stage**	
I	7 (10)
II	50 (73)
III	11 (16)
IV	1 (1)
**Neoadjuvant Treatment**	
Yes	17 (25)
No	52 (75)

## Data Availability

The data presented in this study are available on request from the corresponding author.

## Supplementary Materials

The following are available online at https://www.mdpi.com/2072-6694/13/6/1297/s1, Figure S1: Association of marker expression by tumor-infiltrating T cells and survival.
