# Supplementary material for: LAG-3-Expressing Tumor-Infiltrating T Cells Are Associated with Reduced Disease-Free Survival in Pancreatic Cancer
Source: Cancers (Basel). 2021 Mar 15;13(6):1297. doi: 10.3390/cancers13061297 (PMC7998134; doi:10.3390/cancers13061297)
Supplement: Supplementary file 1 [file cancers-13-01297-s001.pdf]

Supplementary Materials: LAG-3-Expressing Tumor-Infiltrating T Cells are Associated with Reduced Disease-Free Survival in Pancreatic Cancer

Lena Seifert, Ioana Plesca, Luise Müller, Ulrich Sommer, Max Heiduk, Janusz von Renesse, David Digomann, Jessica Glück, Anna Klimova, Jürgen Weitz, Marc Schmitz and Adrian M. Seifert

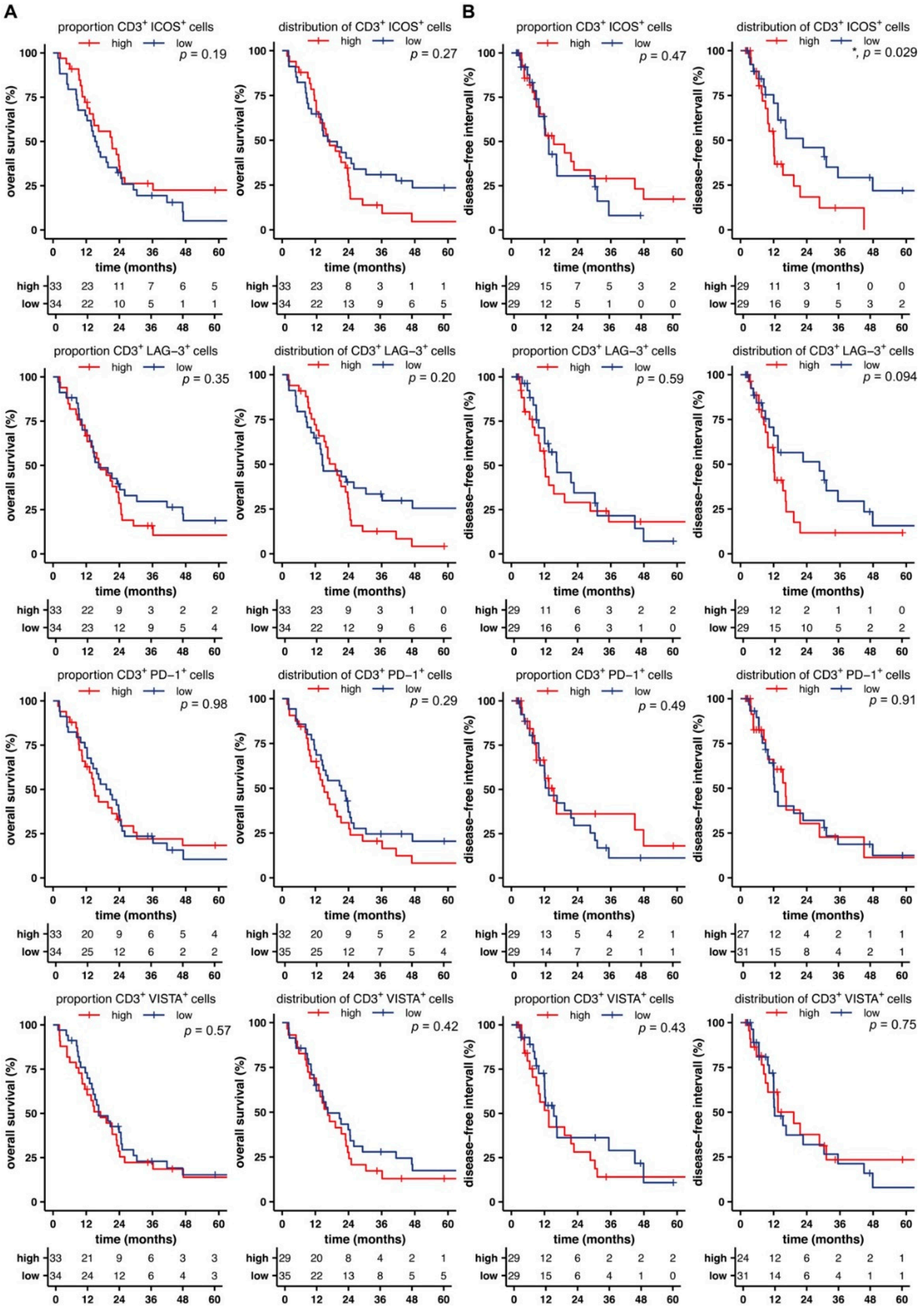

Figure S1. Association between receptor expression by tumor-infiltrating T cells and survival. (**A**) Overall and (**B**) disease-free survival of patients with pancreatic cancer stratified by the median proportion (left) or

distribution (density duct/density stroma; right) of $CD3^+ICOS^+$, $CD3^+LAG\text{-}3^+$, $CD3^+PD\text{-}1^+$, and $CD3^+VISTA^+$ (top to bottom).
